# Optimizing Function and Aesthetics in Kennedy Class 1 Prosthetic Rehabilitation: A Case Report

**DOI:** 10.7759/cureus.66329

**Published:** 2024-08-06

**Authors:** S Vandana Ajay, Surbhi Bayaskar, Tikeshwari Gurav, Aman Thakare, Shubham Deshmukh

**Affiliations:** 1 Department of Prosthodontics, Sharad Pawar Dental College and Hospital, Datta Meghe Institute of Higher Education and Research, Wardha, IND; 2 Department of Oral Medicine and Radiology, Sharad Pawar Dental College and Hospital, Datta Meghe Institute of Higher Education and Research, Wardha, IND; 3 Department of Orthodontics, Sharad Pawar Dental College and Hospital, Datta Meghe Institute of Higher Education and Research, Wardha, IND

**Keywords:** removable prostheses, distal extension, precision attachments, abutment, kennedy’s class i & ii

## Abstract

When dealing with a partially edentulous patient requiring rehabilitation for a distal extension, the situation becomes challenging if the patient is unwilling to consider placing implants or using a removable prosthesis. A patient with partial tooth loss may find it difficult to receive a satisfactory repair, especially if the missing teeth are at the back. The surrounding gum tissue and the underlying bone ridges provide support for dentures that encase natural teeth. During functional activities, these components are frequently exposed to different stresses, which can substantially affect the remaining natural teeth and bone structure. Precision attachments are complex devices made up of two parts: one incorporated into a detachable dental prosthesis and the other fastened to the natural teeth. Their function is to give the prosthetic stability and retention. A fixed partial denture is impractical when there is no distal abutment. However, by offering a combination prosthesis, this difficulty can be solved. Without requiring surgery, this method provides the benefit of a fixed prosthesis. We are presenting a case of management for a 56-year-old patient with unilateral distal extension with a combined prosthesis of acrylic retained by an extra coronal precision attachment system.

## Introduction

One treatment option for Kennedy class 1 or class 2 situations is removable partial dentures (RPDs), the success of which depends on careful and appropriate treatment planning for better appearance and functionality [1]. A fixed partial denture would not be the best option if the posterior abutment tooth is lost. Implant-supported prostheses are another possible treatment option; however, they are not usually practical because of persistent bone resorption and financial constraints. In this situation, a cast or acrylic partial framework is recommended. Patients tend to prefer fixed restorations, such as dental crowns and bridges, because they are clean and nice looking. Nevertheless, these restorations may not be ideal for cases where there has been substantial vertical ridge resorption due to their drawbacks. As such, additional help and thoughts are required to deal with these unique issues. The recommended treatment modality entails using a combination of fixed crown and acrylic partial with precision attachment.

The cast partial dentures are fitted with attachments that are specially made for precision or semi-precision to ensure that they fit securely and retain well. Precision attachments have a lot of benefits in terms of flexibility and adaptability. Better aesthetics, enhanced psychological acceptance of the prosthesis, enhanced stability and retention, decreased bulk and secondary caries incidence, enhanced vertical support, and enhanced stimulation are among the benefits of precise attachments. Its intricate design, intricate manufacturing processes, technical sensitivity, and requirement for proper dental hygiene are its drawbacks. Unfortunately, they have been undermined due to their high prices and misunderstanding of their application [2]. Precision attachment dentures can be classified into two groups: extra-coronal and intra-coronal. These attachments are key in the support of cast partial dentures so that functional and aesthetical replacement of missing teeth is achieved. The new approach of using precision attachment for the management of distal extension aims at creating the most aesthetic and functional achievable partial denture, offering patients a natural, comfortable answer to toothlessness problems. In this case report, we provide a step-by-step account of the fabrication process for a denture retained using an extra coronal precision attachment system for distal extension.

## Case presentation

**Figure 1:**
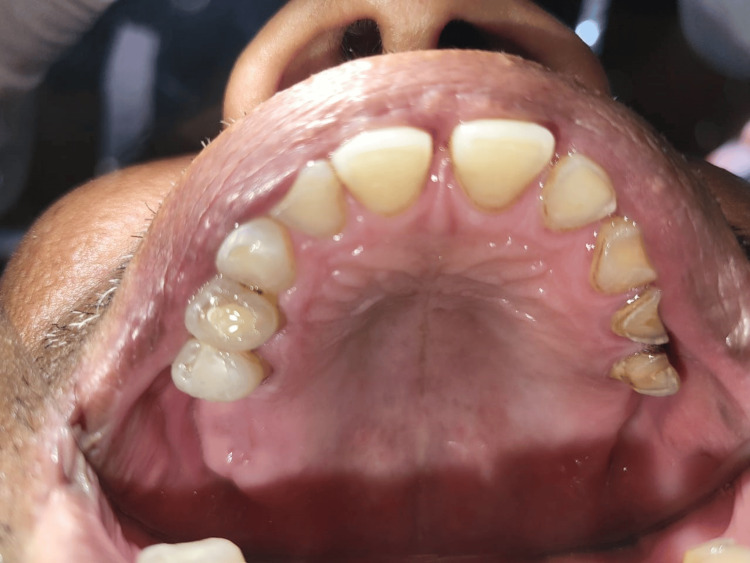
Preoperative intraoral view of maxillary arch showing distal extension.

A 56-year-old male patient reported to the Department of Prosthodontics with the chief complaint of missing teeth in the upper back region of the jaw. The patient gave no relevant medical history. During the intra-oral examination, well-formed upper and lower ridges were observed, and they were in a class 1 relation. It was also noted that the patient was missing the first, second, and third molars on both sides. Tooth-colored restoration was seen with 14 and 15 teeth. Ellis class 2 fracture was also seen with 12 teeth. The remaining teeth in the upper arch were stable in terms of periodontal health. Severe attrition was observed with 23, 24, and 25 teeth. The upper arch was classified as class 1 according to both Applegate's rule and Kennedy's classification, as shown in Figure 1.

After a thorough clinical and radiographic examination, a treatment plan was established. The radiographic examination, which involved taking detailed images of the affected area, did not reveal any signs of periapical lesions, which are abnormalities around the tip of a tooth's root or significant bone loss. This suggests that there are no serious underlying issues in the area that was examined. The plan originally included a combined prosthesis with extra coronal precision attachment for the bilateral distal extension of the maxillary arch. However, the patient expressed that he was not ready to restore both sides, so the treatment plan was modified to replace missing teeth unilaterally. A combined prosthesis with extra coronal precision attachment was then planned for the left distal extension of the maxillary arch. Tooth preparation was performed on 24 and 25 to receive crowns.

**Figure 2:**
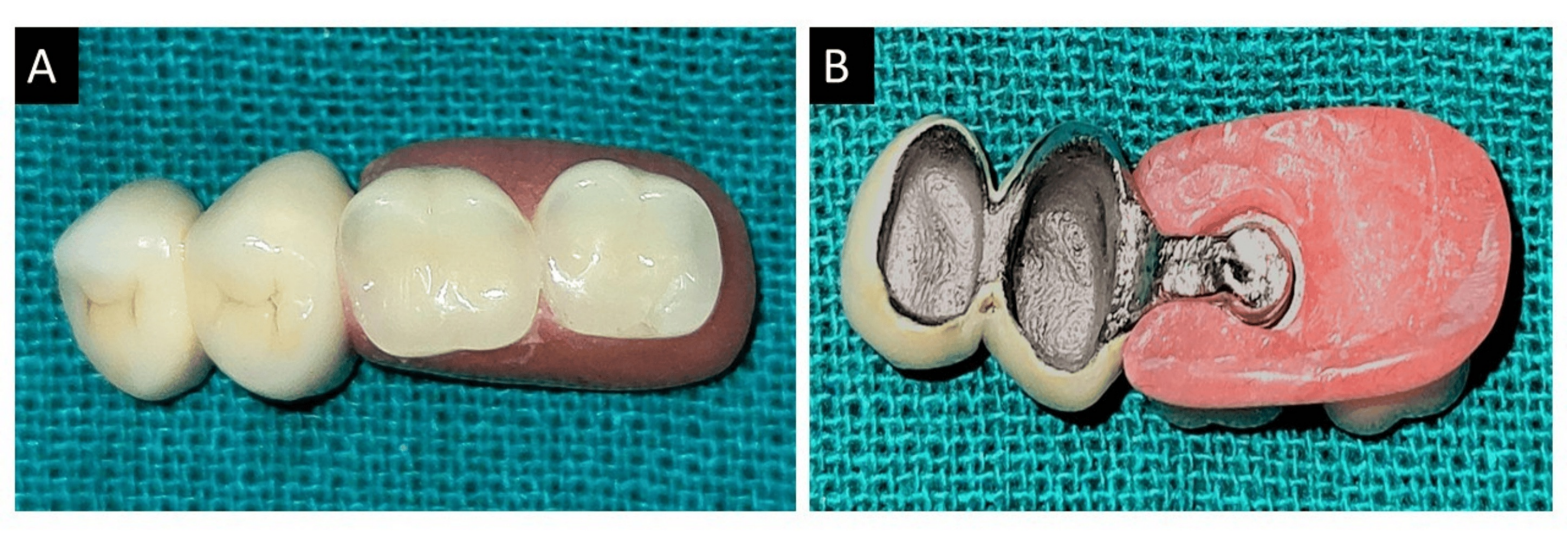
Combined prosthesis. (A) Occlusal view. (B) Inferior view.

**Figure 3:**
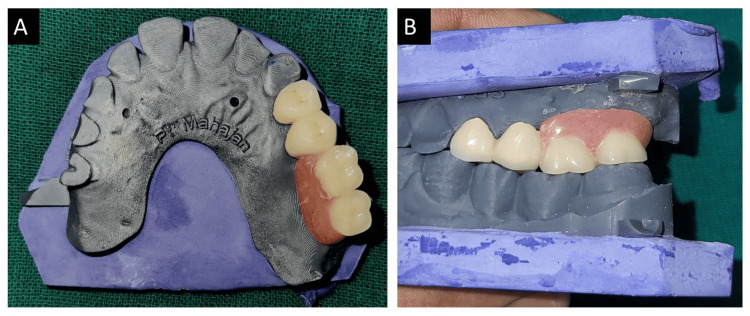
Combined prosthesis in occlusion. (A) Alignment of the prosthesis in an arch. (B) Occlusion on dental casts.

Once the abutments were ready, intra-oral scanning was conducted, and the abutments were temporarily covered. The STL file was sent to the lab, and the casting was completed. The joint crowns were meticulously fabricated in the laboratory with the extra coronal castable precision attachments. To verify the exact fit of the crowns, a meticulous trial was conducted. During this process, ceramic powder was delicately added to the abutments to ensure a seamless finish. The crowns were then subjected to porcelain firing, a meticulous and elaborate process aimed at achieving the desired aesthetic and functional properties. The male part of the fixed component, which was made of matrices, was gently inserted into the patient's mouth. After that, a pick-up imprint was made using polyvinyl siloxanes (PVS) impression material to capture the contours and form of the dental structures precisely. After this, the prosthesis was fabricated, as shown in Figure 2, and the alignment of teeth was examined and confirmed using dental casts to ensure the perfect fit and functionality of the prosthesis, as shown in Figure 3.

**Figure 4:**
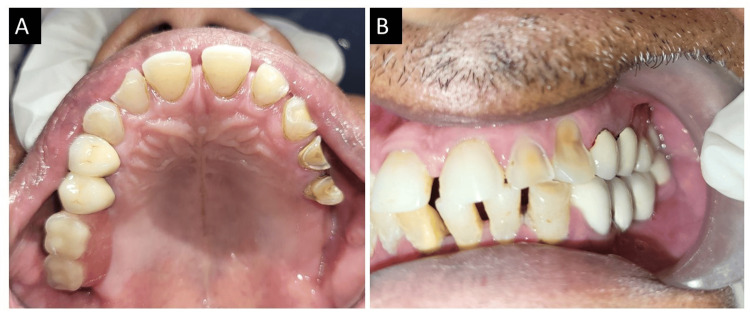
Postoperative intraoral view of combined prosthesis. (A) Intraoral view. (B) In occlusion.

Glass ionomer cement was used to bond the crowns, and an extra coronal castable precision attachment was used to firmly hold the acrylic prosthesis. To verify correct alignment and function, the occlusal contacts were carefully assessed in both centric and eccentric locations, as shown in Figure 4.

The patient was given a follow-up visit for a post-insertion examination one week following the operation. The patient also received comprehensive information on how to maintain good oral hygiene to guarantee the stability and life of the dental treatment.

## Discussion

A precision attachment connection is made up of two or more components. A mechanical connection is made between the two parts. The benefits of both fixed and removable restorations might be combined with prosthetics thanks to these attachments [3]. Early in the 20th century, Dr. Herman Chayes was the first to describe the discovery of attachment [4]. When a patient is not able to get a detachable prosthesis or insert implants, rehabilitation of a distal extension partially edentulous patient becomes difficult.

Due to the lack of a distal abutment, a fixed partial denture is not possible. In such a case, the solution is to provide a combined prosthesis. The advantage of fixed prostheses without the necessity for surgery is a combination prosthesis, in which an acrylic prosthesis is secured to the tooth by an additional coronal connection [3,5]. Improved comfort, fewer postoperative adjustments, and improved aesthetics are only a few advantages of removable prostheses with precise attachments.

Precision attachments play a crucial role in dental prosthodontics, particularly in the areas of esthetic zones, force redistribution, control of loading and rotational forces, segmentation of long-span bridges, and acting as stress breakers in free-end saddles and bridges. It is important to note that precision attachments are not recommended for patients who are sick and elderly, those with severe periodontitis, high rates of tooth decay, inadequate space, or narrow facio-lingual dimensions. The advantages of precision attachments are numerous and significant. They include enhanced esthetics, improved psychological acceptance of the prosthesis, increased retention and stability, reduced bulk, decreased risk of secondary tooth decay, improved vertical support, and better stimulation. However, it is essential to be mindful of the disadvantages as well. Precision attachments are complex in design and fabrication procedures. They are technique-sensitive and require meticulous attention to detail. Additionally, maintaining good oral hygiene is paramount when using precision attachments.

These benefits apply particularly to large edentulous jaws, nonparallel abutments, and distal extension bases [6]. Attachment-supported cast partials protect the abutments, need fewer changes, and offer improved function, comfort, and aesthetics, as several studies have shown. The employment of these attachments in fixed prostheses, implant treatments, and overdentures has a significant impact on the success of prostheses in terms of look, comfort, and utility [7].

A distal extension cast partial denture must be successful in controlling stress on the abutments. The proposed method works better than the traditional prosthesis in several areas [8]. The case studies revealed that the heights of the crowns of the abutments were found to be sufficient for maintaining the connection. Additionally, in some instances, splints were strategically placed in front of the edentulous span to ensure an even distribution of stresses.

Apart from being removable, a cast partial denture also facilitates better oral hygiene maintenance. Consequently, patients have greater stability and satisfaction with attachment-retained partial dentures over the long term as opposed to clasp-retained partial dentures [9,10].

## Conclusions

This case study was meant to demonstrate the conscious decision-making that transpires in the event of the absence of a distal abutment and whether a prosthesis is provided on a detachable or permanent basis. Such situations offer a non-surgical solution to distal extension scenarios by the use of a combined prosthesis. Before the commencement of any treatment, through a thorough evaluation, a treatment planning protocol needs to be based on the patient’s aesthetic preferences and perceptions. The longevity of attachment-retained RPD treatment relies heavily on treatment reinforced by visits every three months and preventive strategies. This is a type of prosthodontic rehabilitation procedure that sheds light on the careful balancing act that needs to be in place through the combinations of existing and derived treatment armamentarium for a successful outcome.

## References

[1] Brown D, Carr A (2011). McCracken's Removable Partial Prosthodontics. https://www.sciencedirect.com/book/9780323069908/mccrackens-removable-partial-prosthodontics#book-description.

[2] Goodkind RJ (1984). Precision attachment removable partial dentures for the periodontally compromised patient. Dent Clin North Am.

[3] Gupta N, Bhasin A, Gupta P, Malhotra P (2013). Combined prosthesis with extracoronal castable precision attachments. Case Rep Dent.

[4] Preiskel HW (1984). Precision attachments in prosthodontics. Int J preventive clin dent res.

[5] Munot VK, Nayakar RP, Patil R (2017). Prosthetic rehabilitation of mandibular defects with fixed-removable partial denture prosthesis using precision attachment: a twin case report. Contemp Clin Dent.

[6] Saneja R, Bhatnagar A, Raj N, Dubey P (2020). Semiprecision attachment: a connecting link between the removable and fixed prosthesis. BMJ Case Rep.

[7] S T, Singh A, Rani P, Prakash J, Bs S, C SG (2023). Prosthodontic rehabilitation with Kennedy's class I and class II using an extended precision attachment: a report of two cases. Cureus.

[8] Kumari KS, K S A (2023). An esthetic approach for rehabilitation of long-span edentulous arch using artificial intelligence. Cureus.

[9] Wahjuni S, Mandanie SA (2017). Fabrication of combined prosthesis with castable extracoronal attachments (laboratory procedure). J Voc Health Stud.

[10] Vaidya S, Kapoor C, Bakshi Y, Bhalla S (2015). Achieving an esthetic smile with fixed and removal prosthesis using extracoronal castable precision attachments. J Indian Prosthodont Soc.

